# Prolonged Anæsthesia from Cocaine

**Published:** 1885-10

**Authors:** 


### CULLINGS FROM CONTEMPORARIES.

### PROLONGED ANÆSTHESIA FROM COCAINE.

ANOTHER very important discovery in the use of that wonderful drug, cocaine, has just been made. Dr. J. Leonard Corning (*N. Y. Medical Journal*) says he was reflecting on the fact that anæsthesia produced by injecting cocaine hypodermically, is of short duration, and soon passes off, and that therefore, in order to keep it up sufficiently long in certain cases, repeated injections have to be made, thus running the risk of too profound constitutional effects, as well as necessitating the use of a considerable amount of the pecious drug. He argued that the reason the anæsthesia passes off, is because the cocaine gets into the circulation, and is not only diluted, weakened, but is washed away, as evidenced by the production of the constitutional effects. He therefore concluded if he could prevent the cocaine from being thus carried away, he not only could economize in the use of the drug, but could prolong the effects of the dose. Accordingly he tried a number of experiments to test his theory. A friend placed himself at the Doctor's disposal for the purpose. Five drops of a four per cent solution were injected in the forearm, below the insertion of the biceps and over the course of the external cutaneous nerve. When anæsthesia was produced, the Doctor passed an Esmarch's tourniquet tightly around the arm above the elbow, thus arresting the circulation. (The radial pulse was obliterated.) The anæsthesia continued, over a considerable area, for forty minutes, when he removed the tourniquet, not, however, because the anæsthesia showed the slightest diminution, for on investigation it proved to be as profound as ever, but on account of the unnecessary tightness of the tourniquet, which caused the gentleman experimented upon considerable inconvenience. In a few minutes after removing the bandage, the anæsthesia began to decline, and was soon entirely lost.

He then exsanguinated the forearm, and applied tourniquet above the elbow, and injected ten drops of the solution. It produced anæsthesia *only at the point where it was introducced,* and was not absorbed—the little blister-like elevations caused by the injection persisting till he, by massage, brought about a kind of mechanical distribution, whereon anæsthesia was induced and persisted till tourniquet was removed at end of forty minutes, during all of which time, and up to taking off the tourniquet, it was profound.

His third experiment consisted in injecting five drops in the neighborhood of the external cutaneous nerve of the forearm as in first experiment, and five or six minutes afterwards, instead of applying the tourniquet immediately, as before, he first exsanguinated the forearm by means of the rubber bandage of Esmarch, "*taking care not to compress the tissues immediately above the point of injection.*" He then applied the tourniquet. The area of anæsthesia was much larger in this instance—following the direction of the nerve some three inches, the greatest width of the zone or area being a little less than an inch, and persisting up to the time of removal of the tourniquet which was this time allowed to remain one hour.

The Doctor summarizes his experiments thus: No. 1 shows that arrest of circulation, after induction of anæsthesia, will prolong it; No. 2 shows that if injection is made after arrest of circulation, the anæsthesia is not diffused; No. 3 proves that if injection is made a few minutes before exsanguination and arrest of circulation, a sufficient amount of saturation of tissue is obtained to expose a large number of nerve filaments to the influence of the anæsthetic.

The advantages are obvious. There is no danger of over dose and alarming constitutional effects. It saves the drug, and relieves the trouble of repeated injections, and prolongs the anæsthesia a sufficient length of time to perform almost any operation. In operations on the extremities it may ultimately obviate the necessity of use of general anæsthetics, while for neuralgias and other disorders of the peripheral nervous system it is indeed a specific for the arrest of pain.

